# Videolaryngoscopy in trauma and cervical immobilization: what should guide our first attempt?

**DOI:** 10.1016/j.bjane.2026.844844

**Published:** 2026-10-13

**Authors:** Liana M.T.A. Azi

**Affiliations:** Universidade Federal da Bahia, Hospital Universitário Professor Edgard Santos, Department of Anesthesiology, Salvador, BA, Brazil

Two recent editorials in the Brazilian Journal of Anesthesiology have addressed some practical concerns regarding intubation: how to describe the view obtained with videolaryngoscopy, and why a better image does not replace training and clinical judgement.^1^^,^^2^ These concerns are particularly relevant when the cervical movement is restricted. The anesthesiologist must obtain a useful view, advance the tracheal tube, and particularly avoid unnecessary movement without prolonging an unsuccessful attempt. These are related tasks, but the success in one does not establish the success in the others.

These outcomes should not be conflated. Videolaryngoscopy may improve intubation success, but whether it also reduces cervical movement and, more importantly, neurological deterioration remains less certain. Current evidence supports its use for the first question more clearly than for the other two. The 2024 multisociety guideline recommends videolaryngoscopy, where possible, in suspected or confirmed cervical spine injury, but cannot recommend a particular device or blade. Importantly, the guideline also states that there are no data showing a greater risk of secondary spinal cord injury with direct laryngoscopy.^3^ So, the preference for videolaryngoscopy should not be presented as a proof of spinal protection.

There is now directly relevant randomized evidence in trauma. In the preplanned trauma analysis of the DEVICE trial, published in 2026, first-attempt intubation succeeded in 151/171 patients allocated to videolaryngoscopy and 114/167 allocated to direct laryngoscopy: 88% versus 68%, an absolute difference of 20 percentage points (95% confidence interval, 11-29). Even so, the severe complications during intubation and in-hospital outcomes did not differ significantly.^4^ This finding supports videolaryngoscopy for emergency intubation after trauma, but it does not establish a mortality benefit or a neurological protection in unstable cervical injury.

This result is also consistent with the National Emergency Airway Registry analysis of 4,449 trauma intubations, which reported first-pass success of 90% with video and 79% with direct laryngoscopy. The association persisted after propensity matching, although residual confounding could be possible.^5^ Earlier results were less favorable. Yeatts and colleagues found no improvement in first-pass success or overall survival with GlideScope, and intubation took longer. A retrospectively identified severe head injury subgroup had more hypoxemia and higher mortality with GlideScope; this remains a cautionary, post hoc finding, not an established contraindication.^6^ Taken together, these findings favor videolaryngoscopy, but they also remind us that the device alone does not determine the success of an intubation.

The presence of a cervical collar in those patients, however, is not a diagnosis of cervical instability. Nor does applying a collar to an elective surgical patient reproduce an emergency trauma airway. In the network meta-analysis of 80 trials involving 8,039 participants, several devices improved first-pass success relative to Macintosh laryngoscopy. The authors nevertheless called for more studies using manual in-line stabilization rather than collar-based models.^7^ Results obtained with a complete collar, a removed anterior piece, or manual stabilization should not be treated as if they were interchangeable.

In this issue, the two new BJAN studies help clarify different parts of this problem. Nguyen and colleagues compared GlideRite rigid and Flex-it articulated stylets with a hyperangulated GlideScope blade in selected elective patients wearing a semi-rigid collar, with the head fixed to the operating table. Among 75 analyzed patients, median intubation duration was 49 seconds with GlideRite and 88 seconds with Flex-it. Protocol-defined failure occurred in 2/37 and 15/38 patients, respectively.^8^ The comparison was between stylets, not between video and direct laryngoscopy. Also, they measured neither cervical movement nor neurological outcomes. Here, the practical lesson is that the stylet is part of the intubation technique, not a minor accessory. In this trial, an articulated design did not improve tube delivery. The magnitude of the failure difference deserves attention, but the trial was not powered specifically for that outcome, and unequal familiarity with the stylets may have influenced performance.^8^ These failure rates should not be transported to trauma patients or interpreted as a universal ranking of stylets. But they do justify training with the precise blade, tube and stylet combination that will be used clinically. In the second manuscript in this issue, Doan and colleagues studied 56 adults undergoing elective cervical spine surgery, comparing hyperangulated ClearVue videolaryngoscopy with Macintosh laryngoscopy under manual in-line stabilization, without a collar. Mean measured C0-C5 angular change was 19.71° versus 26.32°, a difference of 6.61° (95% confidence interval, 2.91-10.31). The C0-C1 difference was also significant, but differences at the other individual segments were not. First-attempt success did not differ significantly. Some participants were classified as having cervical trauma, but this was not an emergency trial in patients with documented unstable injuries; acute trauma with established spinal cord injury was excluded. The study provides useful biomechanical evidence, but its clinical boundaries are important. Images were obtained in the neutral position and at the best glottic view, so they need not capture peak movement during tube advancement. Also, neurological outcomes were not measured.^9^

The movement reduction is not a uniform property of videolaryngoscopy, as Robitaille and colleagues found better glottic visualization with GlideScope but no significant reduction in segmental movement under manual stabilization in 20 patients without cervical pathology.^10^ Different blades, measurement methods and patient populations matter. A difference in degrees cannot yet be translated into an established reduction in spinal cord injury risk. The collar management also deserves more attention than a simple instruction to “keep the neck still”. The 2024 guideline recommends removing a rigid or a semi-rigid collar during intubation attempts, most easily by removing its anterior part; this is a weak recommendation based on low-grade evidence.^3^ In 102 elective patients without cervical spine disease, Jo and colleagues compared Macintosh-style videolaryngoscopy with the posterior collar piece alone versus both pieces. Median intubation time was shorter without the anterior piece, 23 versus 33 seconds, while mean between-group motion differences were approximately one degree and not statistically significant.^11^ The study therefore updates intubating conditions in this model, but it cannot establish the neurological safety of collar removal in an unstable cervical spine.

Flexible bronchoscopic intubation should not be dismissed based on collar studies. Choi and colleagues found higher first-attempt success with Macintosh-style videolaryngoscopy than with flexible fibrescopy in 330 elective cervical surgery patients wearing a collar to simulate difficulty.^12^ That comparison does not answer which awake technique is preferable for a cooperative patient with known instability. In a separate randomized trial of 46 patients with upper cervical instability, Dutta and colleagues found greater C1-C2 movement with awake McGrath intubation than with awake fibreoptic intubation. Neither group developed a new motor deficit, but the small sample cannot establish equivalent neurological safety.^13^ The decision about awake intubation must remain distinct from the choice of laryngoscope after induction.

For Brazilian anesthesiologists, the immediate implication is very practical: a videolaryngoscope intended for trauma should not be a device used only after direct laryngoscopy has failed! Regular use and training should include restricted mouth opening, the chosen stylet, suction, tube advancement and an agreed rescue plan. The guideline explicitly recommends regular videolaryngoscopy training with cervical immobilization and consideration of a stylet or bougie.^3^

Further studies should move beyond reporting only the best glottic view. They should distinguish acute trauma from elective cervical disease, describe injury stability and the exact immobilization technique, and record blade geometry, operator experience, first-attempt success, and physiological complications. Where cervical motion is measured, its timing and segmental distribution should be explicit. Neurological assessments are needed, although uncommon adverse events will require larger multicenter studies. The useful clinical objective is a successful, well-prepared first attempt with no unnecessary movement, and not the best image at any cost.

## Data availability statement

No new data were created or analyzed in this study. Data sharing is not applicable to this article.

## IA disclosure

During the preparation of this manuscript, the author used Grammarly solely for English-language editing, including grammar, spelling, style, and readability. The author reviewed and approved all suggested changes and take full responsibility for the final content of the manuscript.

## Conflicts of interest

The author declares no conflicts of interest.
